# Nanoarchitectured conjugates targeting angiogenesis: investigating heparin-taurocholate acid conjugates (LHT7) as an advanced anti-angiogenic therapy for brain tumor treatment

**DOI:** 10.1186/s40824-023-00420-8

**Published:** 2023-09-18

**Authors:** Hyung Shik Kim, Jae Hak Seol, Hae Hyun Hwang, Dong Yun Lee

**Affiliations:** 1 Department of Bioengineering, College of Engineering, and BK FOUR Biopharmaceutical Innovation Leader for Education and Research Group, Hanyang University, 222 Wangsimni-Ro, Seongdong-Gu, Seoul, 04763 Republic of Korea; 2 Institute of Nano Science and Technology (INST), Hanyang University, Seoul, 04763 Republic of Korea; 3 Institute for Bioengineering and Biopharmaceutical Research (IBBR), Hanyang University, Seoul, 04763 Republic of Korea; 4 Elixir Pharmatech Inc, Seoul, 07463 Republic of Korea

**Keywords:** Glioblastoma, U87MG, Heparin-taurocholate conjugate (LHT7), Anti-angiogenesis, Orthotopic model

## Abstract

**Background:**

Glioblastoma is a highly malignant brain tumor associated with poor prognosis. Conventional therapeutic approaches have limitations due to their toxic effects on normal tissue and the development of tumor cell resistance. This study aimed to explore alternative mechanisms for glioblastoma treatment by targeting angiogenesis.

**Methods:**

The study investigated the anti-angiogenic properties of heparin in glioblastoma treatment. To overcome the limitations of heparin, a heparin-taurocholate conjugate (LHT7) was synthesized by conjugating heparin to taurocholic acid. The study utilized the U87MG human glioblastoma cell line and human umbilical vein endothelial cells (HUVEC) as experimental models. Cell viability assays and sprouting assays were performed to assess the effects of LHT7. Additionally, phosphorylation of angiogenesis-related proteins, such as phospho-ERK and phospho-VEGFR2, was measured. The anti-angiogenic effects of LHT7 were further evaluated using a glioblastoma orthotopic mouse model.

**Results:**

Treatment with LHT7 resulted in a dose-dependent reduction in cell viability in U87MG human glioblastoma cells. The sprouting of HUVEC cells was significantly decreased upon LHT7 treatment. Furthermore, LHT7 treatment led to a decrease in the phosphorylation of angiogenesis-related proteins, including phospho-ERK and phospho-VEGFR2. In the glioblastoma orthotopic mouse model, LHT7 exhibited anti-angiogenic effects, supporting its potential as a therapeutic agent.

**Conclusions:**

The conjugation of heparin and taurocholic acid to create LHT7 offers several advantages over conventional therapeutic approaches for glioblastoma. LHT7 demonstrated anti-angiogenic properties, as evidenced by the reduction in cell viability and inhibition of endothelial cell sprouting. Moreover, LHT7 modulated the phosphorylation of angiogenesis-related proteins. These findings suggest that LHT7 holds promise as a medication for glioblastoma treatment, offering potential implications for improving patient outcomes.

**Graphical Abstract:**

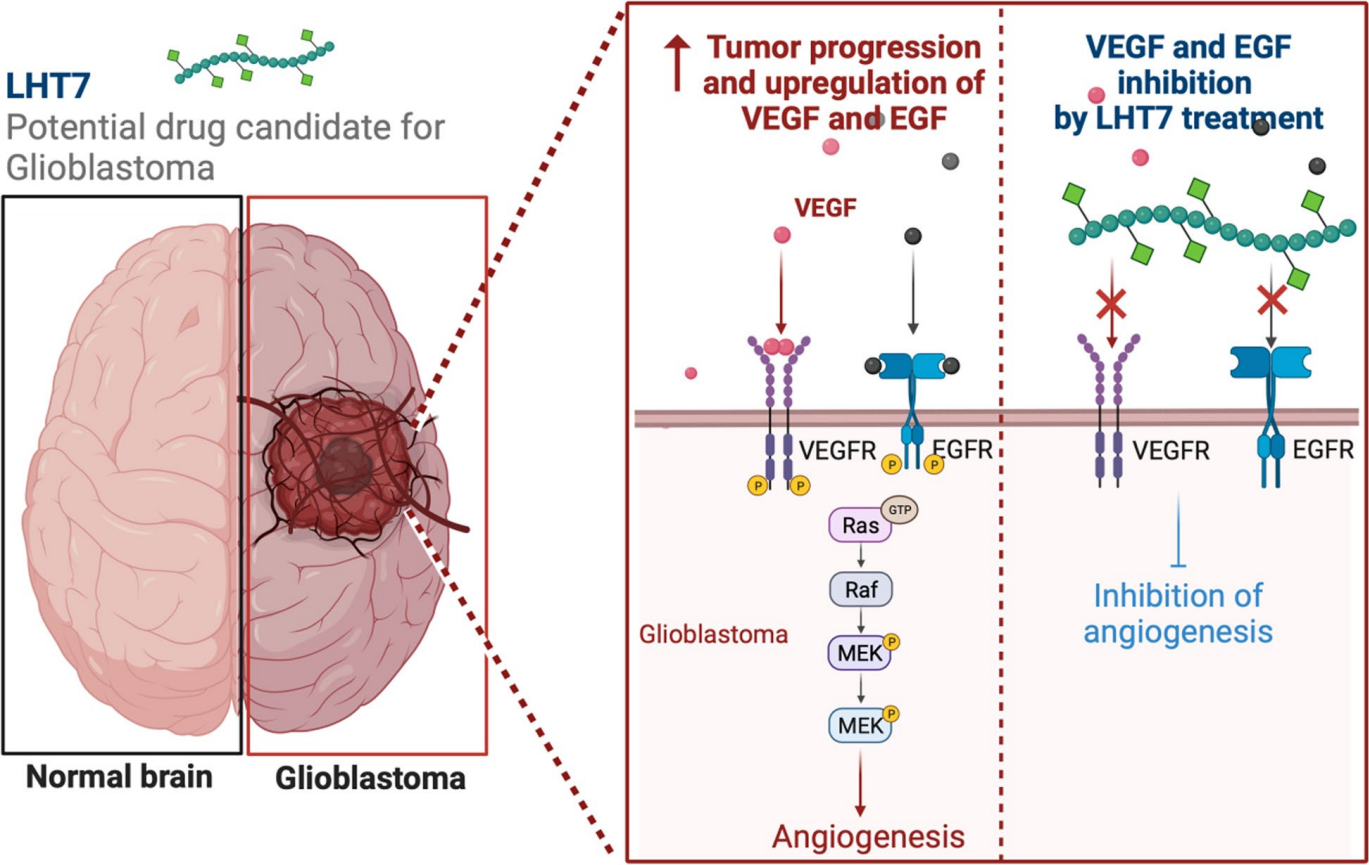

## Background

Glioblastoma is a disease of the central nervous system that is classified as grade IV according to WHO classification [1, 2]. Several chemotherapy agents including temozolomide, 1,3-bis(2-chloroethyl)-1-nitrosourea (BCNU), vincristine, and irinotecan are widely used to treat glioblastoma along with surgical resection and radiotherapy [3]. Among the various therapeutic agents employed for glioblastoma, temozolomide has emerged as a widely used chemotherapy agent. This alkylating agent induces apoptosis through methylation of the O^6^, N^7^, and N^3^ positions of DNA. However, tumor cells can develop temozolomide resistance via DNA damage repair systems such as the O^6^-methylguanine-DNA methyltransferase (MGMT) repair mechanism and the DNA mismatch repair (MMR) system [4]. Furthermore, because of the lack of specificity to cancer, normal tissue is also affected by temozolomide treatment.

Brain tumors acquire oxygen and nutrients through tumor-induced angiogenesis; therefore, anti-angiogenesis is an established strategy for treatment of glioblastoma with high vascularity and high malignancy [5]. Among the many angiogenic factors, such as vascular endothelial growth factor (VEGF), platelet-derived growth factor (PDGF), and fibroblast growth factor-basic (b-FGF), VEGF is a key regulator of angiogenesis [6]. There are several commercially available anti-angiogenic agents such as Avastin, a monoclonal antibody that targets VEGF; Nexavar, which comprises two kinase inhibitors; Sutent, which targets VEGF receptor 2; and Votrient, which consists of three kinase inhibitors [7]. However, because of increasing resistance to Avastin, glioblastoma patients receiving it as treatment have a poor prognosis [8]. Also, it was reported that alternative angiogenic factors upregulate and reactivate angiogenesis after Avastin treatment [9]. Given the limitations and challenges associated with current anti-angiogenic treatments, there is need to develop new anti-angiogenic drugs that downregulate various angiogenic factors while minimizing side effects.

Heparan sulfate occurs in the extracellular matrix and is responsible for growth factor regulation via binding with VEGF and bFGF [10]. Highly sulfated glycosaminoglycan (HSGAG), unfractionated heparin (UFH), and low-molecular-weight heparin (LMWH), which have similar structures to that of heparan sulfate, were originally regarded as anti-coagulators for thromboembolism in cancer patients. This anti-coagulation effect is initiated with antithrombin-heparin complexation, which is mediated by the high-affinity pentasaccharide sequence of heparin [11, 12]. The interaction between heparin and antithrombin is caused by the physicochemical properties of heparin such as sulfation pattern, charge distribution, and molecular size. Heparin, owing to its diverse physicochemical properties, has the capacity to interact with various growth factors that are involved in angiogenesis, exhibiting anti-angiogenic properties. However, unmodified heparin, despite its therapeutic potential, has limited clinical efficacy due to its short half-life and unfavorable side effects, including hemorrhage.

Several chemically modified heparins have been developed to address the limitations of traditional heparin. These modified heparins, including heparin-deoxycholic acid conjugate and folate-heparin-lithocholate derivatives, reduce the risk of hemorrhage and have anti-angiogenic and anti-metastatic effects compared to heparin alone. Among them, bile acid-acylated heparin derivatives resulted in a higher anti-cancer effect than heparin or LMWH [13–15]. LMWH was conjugated with the bile acid taurocholate, and this heparin-taurocholate conjugate (LHT) was constructed by amide bonding between the carboxyl group of LMWH and the amine group of N-taurocholylethylene diamine. Among the various types of LHT, LHT7 exhibited 12.7% anticoagulation activity compared to that of LMWH, reducing hemorrhage risk [16]. Moreover, due to the taurocholate, LHT7 has a longer half-life (> 4 h) than heparin (< 30 min) [17] and attenuates tumor growth with anti-angiogenic effects through inhibition of multiple stages of angiogenesis that are mediated by VEGF, FGF2, and PDGF-B. For these reasons, LHT7 is a promising potential anti-angiogenesis agent for highly vascularized tumor treatment. LHT7 blocks VEGF-VEGFR binding by neutralizing VEGF and dephosphorylating VEGFR [18]. However, the sub-molecular pathways related to angiogenesis after VEGFR phosphor/dephosphorylation induced by LHT7 remain unknown. Furthermore, it is imperative to assess the impact of LHT7 treatment on the growth of glioblastoma multiforme (GBM) tumors in an orthotopic animal model. In pursuit of these objectives, this study aims to evaluate the anti-tumor effects of LHT7 using an orthotopic mouse model of glioblastoma. Additionally, we seek to elucidate the subcellular signaling pathways involved in the anti-angiogenic therapy mediated by LHT7. By investigating these aspects, we aim to enhance our understanding of LHT7's potential as a therapeutic agent for glioblastoma and shed light on its mechanisms of action at the subcellular level.

## Materials and methods

### Cell lines and animals

All experiments were carried out using human umbilical vein endothelial cells (HUVEC; LONZA, Morristown, NJ, USA) and a human glioblastoma cell line (U87MG; Korean Cell Line Bank, Seoul, Korea) derived from human brain cancer. HUVECs (passage number 4 to 6) were cultured using endothelial growth medium (EGM-2 bullet kit; LONZA), and U87MG was cultured using Dulbecco’s Modified Eagle’s Medium (DMEM; GenDEPOT, Katy, TX, USA) containing 10% fetal bovine serum (FBS; GenDEPOT) and 1% antibiotics in standard culture conditions of 37℃ and 5% CO_2_. In vivo experiments were carried out using five- to seven-week-old male BALB/C nude mice (Nara-Bio Company, Seoul, Korea) and male Sprague–Dawley (SD) rats (Nara-Bio Company). All animals were housed in specific pathogen-free (SPF) conditions and maintained under the guidelines of the Institutional Animal Care and Use Committee (IACUC) at Hanyang University.

### LHT7 preparation

LHT7 preparation was described previously [18]. Briefly, taurocholic acid sodium salt (TCA; Sigma-Aldrich, St. Louis, MO, USA) was mixed with N,N-dimethylformamide (DMF; Sigma-Aldrich), and trimethylamine (Sigma-Aldrich) and 4-nitrophenylchloroformate (4-NPC; Sigma-Aldrich) were added, followed by filtration. The resulting TCA-NPC was mixed with DMF and dissolved in ethylenediamine solution. The reaction yielded TCA-NH_2_. To activate the carboxylic acid group of low-molecular-weight-heparin (LMWH; GSK, Middlesex, UK), 1-ethyl-3-(3-dimethylaminopropyl) carbodiimide hydrochloride (EDC; Sigma-Aldrich) was added, followed by TCA-NH_2_ solution.

### Cell viability assay

2-(2-Methoxy-4-nitrophenyl)-3-(4-nitrophenyl)-5-(2,4-disulfophenyl)-2H-tetrazolium (CCK-8; Dojindo, Kumamoto, Japan) was used for the cell viability assay. HUVEC and U87MG cells were seeded in 96-well plates at a density of 5 × 10^3^ cells/well and were incubated for 24 h in a CO_2_ incubator. For viability measurement, incubated U87MG cells were washed twice with phosphate-buffered saline (PBS) and then treated with 10 ng/mL EGF (PEPROTECH, Cranbury, NJ, USA) or 10 ng/mL VEGF_165_ (PEPROTECH) for 24 h or with various concentrations of LHT7 for 0 and 24 h at 37 °C and 5% CO_2_ condition. After treatment, GF- or LHT7-containing medium was removed, and the cells were washed with PBS. After that, U87MG cells were treated with culture medium and CCK-8 (10% of medium volume) for 4 h at 37℃ and 5% CO_2_. The absorbance of the orange-colored medium was measured with a micro-well plate reader at 450 nm and quantified by Sigma Plot 10.0 (Systat Software, San Jose, CA, USA). In the HUVECs, the overall procedures were the same as those with U87MG viability measurements, except for GF and LHT7 co-treatment (24 h).

### Western blot

The Western blot procedure has previously been described [16]. Briefly, when U87MG and HUVEC cell confluence reached 70–80% of the cell culture dish area, the cells were washed with PBS, followed by addition of starvation medium (DMEM or EBM-2 + 0.5% FBS) for 12 h (U87MG) and 4 h (HUVECs). After starvation, the media was changed to that containing 50 ng/mL growth factor with or without 100 μg/mL LHT7 for 1–2 h. The cells were lysed with protease and phosphatase inhibitor cocktail (Thermo Scientific, Waltham, MA, USA) containing RIPA buffer (Thermo Scientific), followed by sonication. The remaining component was removed by centrifugation (14,000 rpm, 4℃, 15 min), and protein concentration was determined by BCA assay. The supernatant was mixed with 4 × sample buffer (Bio-Rad, Hercules, CA, USA) followed by boiling at 96℃ for 5 min.

Whole-cell protein (30 μg) was loaded in 10% polyacrylamide gel, and SDS-PAGE was carried out for 2 h. Separated proteins were electroblotted onto the PVDF membrane (Millipore, Danvers, MA, USA) for 2–3 h. After that, the PVDF membrane was blocked for 1 h with 5% skim milk or 5% BSA solution, followed by treatment with anti-FAK, anti-phosphorylated FAK antibody (SAB, SD, USA); anti-AKT, anti-phosphorylated AKT antibody (Cell Signaling Technology, Danvers, MA, USA); anti-ERK, anti-phosphorylated ERK, anti-phosphorylated VEGFR2 antibody (Thermo Scientific); anti-VEGFR2, anti-EGFR, anti-phosphorylated EGFR; and anti-cyclin D1 antibody (Abcam, Cambridge, UK) for 2 h. β-Actin was used as a loading control, and the band was detected using the ECL system (Thermo Scientific). Band intensity on Western blot images was measured using Image J and quantified by Sigma Plot 10.0 (Systat Software).

### VEGF ELISA

To confirm the amount of VEGF expression of U87MG under LHT7, a commercial human VEGF ELISA kit (Koma Biotech, Seoul, Korea) was used. Cells were treated with 100 μg/mL LHT7 for 24 h, followed by collection of cells with RIPA buffer for cell lysis. After that, the remaining step was carried out according to the manufacturer’s instructions. Finally, absorbance was measured using a micro-well plate reader at 450 nm and quantified by Sigma Plot 10.0 (Systat Software).

### Rat aortic ring assay

The rat aortic ring assay was performed according to a previous method with some modification [19]. The aortas of SD rats were cut into small rings with 1–1.5 mm thickness. Forty-eight-well plates were pre-coated with 100 μL Matrigel (BD Bioscience, San Jose, CA, USA) and incubated at 37℃ for polymerization. The aortic ring was seeded upon pre-coated Matrigel and over-coated by 100 μL Matrigel. Subsequently, the coated aortic ring was treated with 10 ng/mL VEGF_165_ with or without various concentrations of LHT7 for a final volume of 700 μL. The media was changed every other day. Six days after treatment, sprouting microvessels were captured using inverted microscopy and quantified by Image-Pro Plus 7.0 software (Media Cybernetics Inc., Rockville, MD, USA) and Sigma Plot 10.0 (Systat Software).

### Orthotopic glioblastoma mouse model and animal experiments

To construct the orthotopic glioblastoma mouse model, intracranial injection was carried out by an established method with some modification [20]. The U87MG cell line was suspended with PBS not containing antibiotics. Five- to seven-week-old male nude mice were anesthetized with 3% isoflurane (HanaPharm, Seoul, Korea) and fixed by ear bar in the stereotaxic instrument (Stoelting Co, Wood Dale, IL, USA). After the mouse was anesthetized, the scalp was removed in the surgical position, and a small hole was created 2-mm right lateral and 2-mm posterior from bregma with a sterile drill. Next, 1 × 10^6^ U87MG cells in 8 μL PBS were loaded into a 26G Hamilton syringe (Hamilton Company, Reno, NV, USA) that was then placed in the stereotaxic apparatus. Cells were injected at a rate of 1 μL/min at 3-mm deep, followed by a 3-min wait to prevent overflow. After injection, the hole was sealed with bone wax, and the scalp was closed with suturing.

The mice were randomly divided into a control group and an LHT7-treated group. The LHT7-treated group for the prevention test received 5 mg/kg LHT7 every other day for 1 month after the U87MG injection. The short-term LHT7-treated group received 5 mg/kg LHT7 every other day for 2 weeks, starting 1 month after the U87MG injection. The control groups were treated with PBS for the same period as the associated experimental group.

### Histology and immunofluorescence

After the animal experiment ended, all mice were perfused with 4% paraformaldehyde solution. Removed mouse brains then were fixed with 4% paraformaldehyde solution for 1 week at 4℃. Positions 1–2 mm lateral from the U87MG injection point were cut and flushed with tap water overnight, followed by embedding with paraffin. Brain slices with 4–6-μm thickness were obtained using a paraffin microtome (Leica Microsystems, Wetzlar, Germany).

To visualize the brain tumors, sliced brain samples were stained with 0.1% cresyl violet (Sigma Aldrich) solution. For sample mounting, histological mounting medium (Fisher Scientific, NJ, USA) was used. The brain tumor volume was calculated using the volume formula (major axis) x (minor axis)^2^ × 0.52. Images were captured using an EOS 650D (Canon, Tokyo, JAPAN) and quantified by Image J and Sigma Plot 10.0 (Systat Software).

The remaining brain samples were used for immunofluorescence analysis. Serum blocking was carried out with 20% goat serum in PBS-T at room temperature for 30 min under humidified conditions (to prevent drying). To confirm tight junction degradation, blocked brain samples were treated with rabbit anti-occludin antibody (Invitrogen, Waltham, MD, USA) at room temperature for 1 h and washed with PBS-T. Occludin-blotted brain samples were treated with secondary antibody (AlexaFluor 488 goat anti-rabbit antibody, Invitrogen) at room temperature for 1 h. Last, the brain samples were washed with PBS and mounted with DAPI mounting medium (Vector Laboratories, Newark, CA, USA). To evaluate the anti-angiogenic and anti-proliferation effects of LHT7, rat anti-CD34 antibody (Abcam, Waltham, MA, USA) and rabbit anti-KI67 antibody (Abcam) were used. The overall steps were similar to those above, except for the secondary antibody FITC-conjugated goat anti-rat antibody (Abcam). All images were captured using fluorescence microscopy (Eclipse TE2000-S) and quantified using Image J and Sigma Plot 10.0 (Systat Software).

## Results

### Inhibitory effect of LHT7 on U87MG proliferation through ERK dephosphorylation

**Fig. 1:**
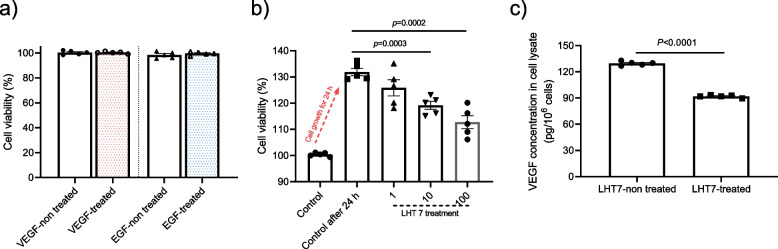
Anti-proliferation effect of LHT7 on U87MG. **a** Cell viability of U87MG in the presence or absence of VEGF (10 μg/mL) and EGF (10 μg/mL), respectively. Data are expressed as mean ± SEM (*n* = 5). **b** Cell viability of U87MG with LHT7 treatment in the concentration range of 1–100 μg/mL. Data are expressed as mean ± SEM (*n* = 5). **c** VEGF concentration in U87MG cell lysate after 24 h treatment with LHT7 (100 μg/mL). Data are expressed as mean ± SEM (*n* = 5)

**Fig. 2:**
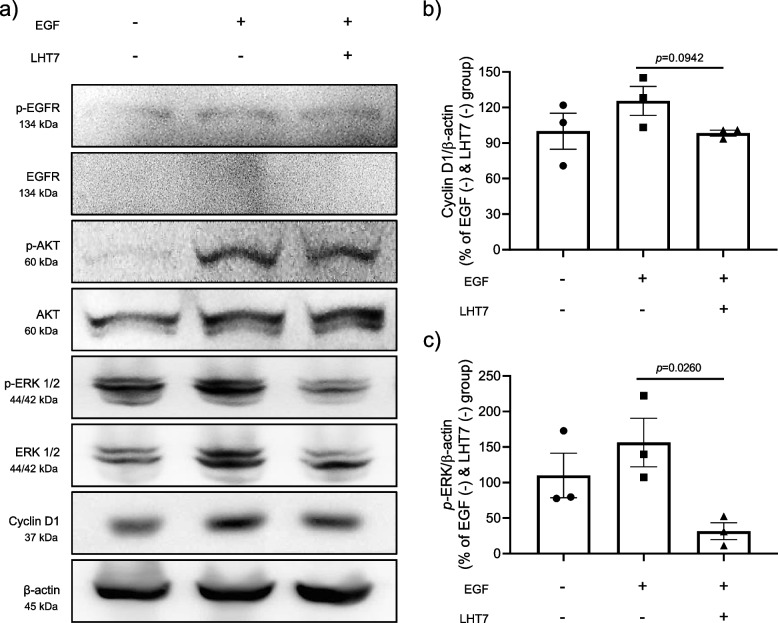
Attenuation of U87MG cell proliferation through ERK dephosphorylation. **a** Western blot result of U87MG. **b** Densitometric results of the related bands are expressed as relative optical band density corrected by that of total Cyclin D1 proteins as a loading control and normalized against the untreated control (EGF (-) and LHT7 (-) group). Data are expressed as mean ± SEM (*n* = 3). **c** Densitometric results of the related bands are expressed as the relative optical band density corrected by that of total *p-*ERK proteins as a loading control and normalized against the untreated control (EGF (-) and LHT7 (-) group). Data are expressed as mean ± SEM (*n* = 3)

In the tumor microenvironment, several growth factors such as EGF and VEGF are overexpressed. Thus, proliferation of U87MG was assessed in response to treatment with EGF (10 ng/mL), which plays an essential role in glioblastoma metastasis, and VEGF (10 ng/mL), which acts as a major angiogenic factor (Fig. 1a). Extracellular application of each growth factor did not influence U87MG proliferation, which was significantly decreased by LHT7 in a dose-dependent manner (treatment with 1, 10, and 100 μg/mL of LHT7 decreased proliferation by 3.85, 9.37, and 14.27%, respectively) (Fig. 1b). In this regard, it is worth noting that chemically modified heparin has been reported to exhibit reliability in terms of cell viability even at concentrations as high as 1000 μg/mL. This observation suggests that the inhibition of the VEGF signaling pathway, encouraged by the use of modified heparin, could potentially contribute to the suppression of tumor growth. Indeed, LHT7 treatment (100 μg/mL for 24 h) reduced the expression of VEGF in U87MG cell lysate by 70.89% compared to the LHT7 untreated group (Fig. 1c). Attenuation of VEGF expression by LHT7 treatment is important because it represents disruption of signaling pathways involved in glioma cell proliferation, migration, and survival [21]. Next, Western blotting was conducted to decipher the mechanism of LHT7 in inhibiting the proliferation of U87MG cells through VEGF down-regulation (Fig. 2a). U87MG cells were treated with 10 ng/mL EGF (positive control) and 100 μg/mL LHT7 because the VEGF and EGF pathways are closely related, sharing downstream signaling pathways in tumor cell proliferation. Furthermore, EGF, a key EGFR ligand, is one of the many growth factors that drive VEGF expression [22]. In phosphorylated EGFR, the slight increase in band intensity induced by EGF was decreased by LHT7 treatment (Fig. 2b). Phosphorylated AKT, a marker of cell proliferation and apoptosis, and cyclin D1, a marker of cell cycle progression (G1/S transition), were decreased by LHT7, but the change was not statistically significant. Most notably, LHT7 reduced the band intensity of phosphorylated ERK, which reflects proliferation and VEGF expression, by 124.85% (Fig. 2c). These findings show that LHT7 inhibits U87MG cell growth and VEGF expression primarily through ERK dephosphorylation.

### Anti-proliferative action of LHT7 on HUVECs via dephosphorylation of the ERK signaling pathway

**Fig. 3:**
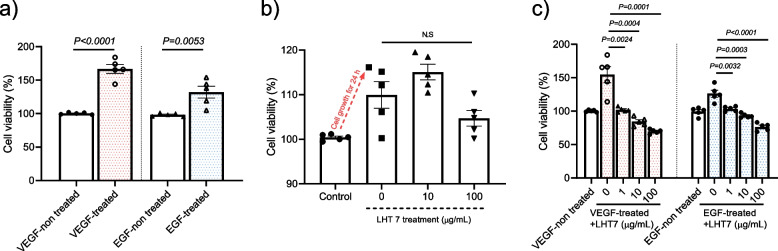
Anti-proliferation effect of LHT7 on HUVEC cells. **a** Cell viability of HUVECs in the presence or absence of VEGF (10 μg/mL) or EGF (10 μg/mL). Data are expressed as mean ± SEM (*n* = 5). **b** Cell viability of U87MG with LHT7 treatment in the concentration range of 0–100 μg/m. Data are expressed as mean ± SEM (*n* = 5). **c** Cell viability of HUVECs in the presence of growth factors (VEGF or EGF) and LHT in the concentration range of 0–100 μg/mL. Data are expressed as mean ± SEM (*n* = 5

To validate the anti-proliferative action of LHT7 on HUVECs, we performed a cell viability experiment under the same conditions as used for U87MG. HUVECs were treated with EGF (10 ng/mL) or VEGF (10 ng/mL) for 24 h. Contrary to the pattern in U87MG, extracellular treatment with growth factors enhanced proliferation by 61.06% and 33.63% with VEGF and EGF, respectively (Fig. 3a). LHT7 had a marginal inhibitory effect on HUVEC proliferation, and the treatment with 100 μg/mL LHT7 did not significantly decrease cell viability compared to the 0 μg/mL treatment group (Fig. 3b). Since tumor angiogenesis is primarily caused by induction of an angiogenic switch (from a quiescent state to a proliferative vasculature) through secretion of angiogenesis-stimulating growth factors, HUVECs were treated with 10 ng/mL VEGF or EGF [23]. Both VEGF and EGF treatment drastically upregulated the proliferation of HUVECs (Fig. 3c). On the other hand, co-treatment of VEGF and 1, 10, and 100 μg/mL LHT7 reduced proliferation by 39.29, 59.21, and 76.28%, respectively. This same co-treatment with EGF significantly decreased proliferation by 20.11, 29.81, and 45.95%, respectively. Taken together, these results indicate that LHT7 efficiently counteracts tumor angiogenic signaling pathways driven by bioactive growth factors involved in neovascularization.

**Fig. 4:**
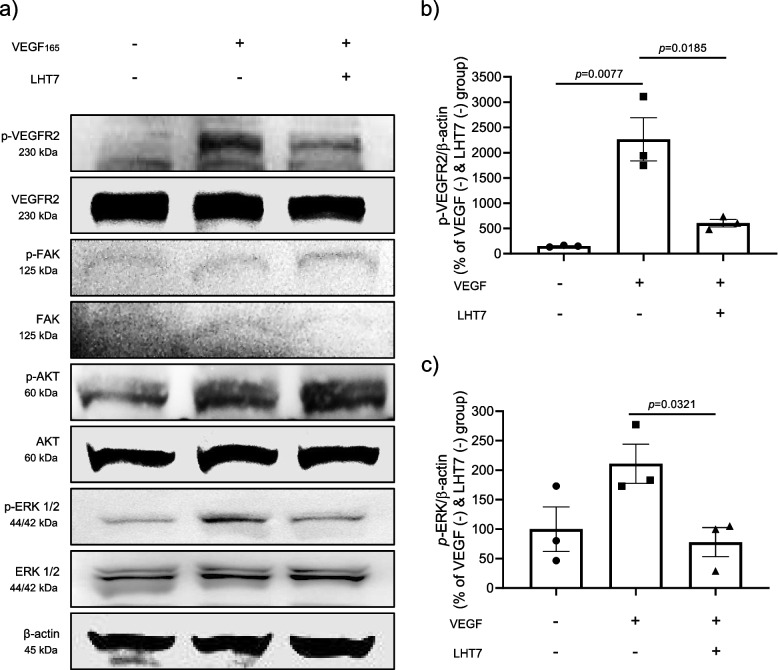
Attenuation of HUVEC cell proliferation through ERK dephosphorylation. **a** Western blot result of HUVECs. **b** Densitometric results of the related bands are expressed as the relative optical band density corrected to that of total *p-*VEGF proteins as a loading control and normalized against the untreated control (VEGF (-) and LHT7 (-) group). Data are expressed as mean ± SEM (*n* = 3). **c** Densitometric results of the related bands are expressed as the relative optical band density corrected to that of total *p-*ERK proteins as a loading control and normalized against the untreated control (VEGF (-) and LHT7 (-) group). Data are expressed as mean ± SEM (*n* = 3)

VEGF is crucial for formation of tumor vasculature and ensuing metastasis in pathological angiogenesis, such as glioblastoma. Induction of a crucial component of the VEGF signaling cascade commences with the interaction of VEGF with its receptor, vascular endothelial growth factor receptor type 2 (VEGFR2), in conjunction with its co-receptor, neutrophilin-1 (NRP1), on the surface of endothelial cells. This initial step is instrumental in the activation of downstream signaling pathways that ultimately promote angiogenesis, vasculogenesis, and endothelial cell proliferation, playing a pivotal role in various physiological and pathological processes [24]. To investigate the potential effects of LHT7 on the proliferation of endothelial cells via VEGF-mediated pathways in an in vitro setting, western blot analysis was conducted using HUVECs (Fig. 4a). Initiation of the angiogenic signaling pathway was elicited by treatment of HUVECs with 10 ng/mL VEGF, while the inhibitory effect of LHT7 was evaluated through treatment with 100 μg/mL VEGF. The results revealed a significant decrease in the intensity of the phosphorylated bands of both VEGFR2 and ERK, with reductions of 1661.86% and 133.17%, respectively (Fig. 4b and c). Consistent with the data obtained from U87MG cells, there was no significant decrease in phosphorylation of AKT, a marker of cell survival, or FAK, a marker of cell migration. Based on the findings, LHT7 also suppresses the proliferation of HUVECs, primarily through dephosphorylation of the ERK signaling pathway.

### LHT7 suppresses VEGF-driven angiogenesis in an ex vivo setting

**Fig. 5:**
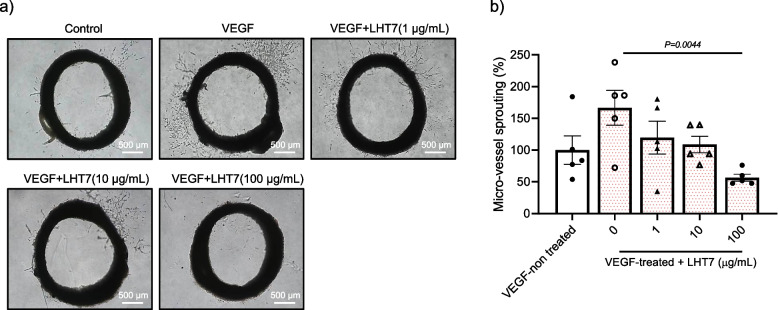
Ex vivo anti-angiogenic effect analysis. **a** Rat aorta ring assay in the presence of VEGF and LHT in the concentration range of 0–100 μg/mL. **b** Quantification analysis for micro-vessel sprouting. Data are expressed as mean ± SEM (*n* = 5)

To investigate the potential anti-angiogenic properties of LHT7 in a complex milieu of interactions among endothelial cells, progenitor cells, and diverse angiogenic factors, aortic rings procured from rats were subjected to LHT7 treatment for 6 days [25]. This experimental design was intended to simulate a physiological environment, allowing a more comprehensive assessment of LHT7's ability to modulate angiogenesis, a crucial process underlying numerous physiological and pathological conditions. The experiment involved treating a rat aortic ring with 10 ng/mL VEGF and then exposing it to different concentrations of LHT7 (1, 10, and 100 μg/mL). The treated aortic ring was observed under a microscope to study the effects of LHT7 concentration (Fig. 5a). Following 6 days of treatment, the number of sprouted microvessels decreased in a dose-dependent manner with increasing concentration of LHT7, with the most pronounced reduction of 109.89% observed at 100 μg/mL (Fig. 5b). These findings suggest that LHT7 exerts potent anti-angiogenic effects, which could be attributed to its ability to modulate the intricate interplay among endothelial cells, progenitor cells, and angiogenic factors. Such observations could pave the way for the development of novel therapeutic strategies targeting aberrant angiogenesis in various pathological conditions.

### Anti-tumor and anti-angiogenesis effects of LHT7 in a glioblastoma orthotopic mouse model

**Fig. 6:**
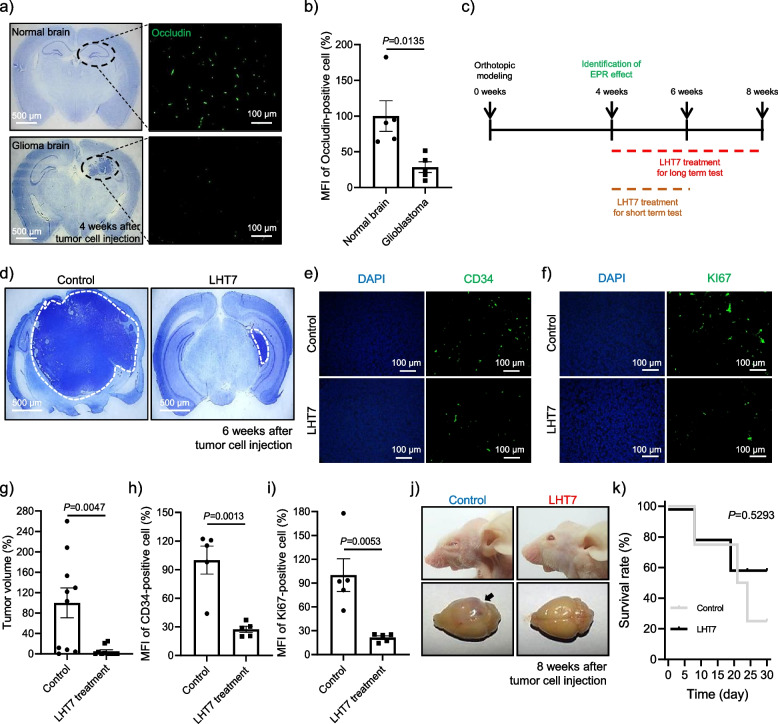
In vivo evaluation of treatment outcomes in an orthotopic glioblastoma mouse model. **a** Immunofluorescence results of tumor-bearing areas stained with anti-occludin antibody. **b** Mean fluorescence intensity (MFI) of occludin-positive cells. Data are expressed as mean ± SEM (*n* = 5). **c** Schematic diagram of the treatment plan. **d** Nissl staining results. White dashed lines represent the glioblastoma area of the whole brain. **e** Immunofluorescence results of tumor-bearing areas stained with anti-CD34 antibody followed by DAPI-nucleus counterstaining. **f** Immunofluorescence results of tumor-bearing areas stained with anti-KI67 antibody followed by DAPI-nucleus counterstaining. **g** Quantification of tumor volume after treatment. Data are expressed as mean ± SEM (*n* = 13). **h** MFI of CD34-positive cells. Data are expressed as mean ± SEM (*n* = 5). **i** MFI of KI67-positive cells. Data are expressed as mean ± SEM (*n* = 5). **j** Representative image of mouse and brain after treatment. The black arrow indicates the glioblastoma-bearing brain. **k** Assessment of 30-day survival rate. Data are expressed as mean ± SEM (*n* = 13)

During tumorigenesis, explosive induction of abnormal angiogenesis occurs when the volume of the tumor reaches 1 mm^3^ [26], and the tight junction integrity of the blood–brain barrier is compromised by both tumor-induced pressure and factors secreted from the tumor [27–31]. To evaluate the potential of LHT7 to reach the tumor site via disrupted tight junctions, we first investigated the presence of an enhanced permeability and retention (EPR) effect in an established orthotopic glioblastoma xenograft mouse model. Four weeks after intracranial injection of U87MG cells to establish a 1 mm^3^ tumor volume, degradation of tight junctions was confirmed through assessment of occludin as a tight junction marker (Fig. 6a) [32]. The immunostaining results revealed a significantly reduced number of occludin-positive cells in the brain with glioma compared to the normal brain, with a decrease of 71.60% (Fig. 6b). This phenomenon offers a potential opportunity for therapeutic agents such as LHT7 to accumulate within brain tumors, enabling more targeted and effective treatment strategies for brain tumor.

Next, we sought to determine the efficacy of LHT7 in inhibiting tumor progression in an orthotopic murine model of glioblastoma. Our in vitro and ex vivo experiments established the effectiveness of LHT7 at concentrations from 10 to 100 μg/mL in inducing anti-angiogenic and anti-proliferative responses in tumor cells. Additionally, a prior study demonstrated optimal outcomes with a regimen of intravenous 5 mg/kg LHT7 every other day. Therefore, mice were subjected to a regimen of intravenous 5 mg/kg LHT7 (calculated based on an estimated blood volume of 80 mL/kg in mice) every day for 2 weeks, commencing 1 month after intracranial injection of U87MG cells (Fig. 6c). At 6 weeks post-tumor induction, Nissl staining revealed a significantly reduced volume of glioblastoma in the LHT7-treated group (12.22 mm^3^) compared to the control group (57.55 mm^3^) (Fig. 6d and g). To corroborate the anti-angiogenic basis of the anti-tumor effect of LHT7, the brain sections of treated mice were immuno-stained using antibodies specific for vessel detection (CD34) and proliferation markers (KI67) (Fig. 6e and f). The results revealed a decrease in the mean fluorescence intensity (MFI) of CD34-positive cells in the LHT7-treated group compared to the control group, with a 72.57% reduction observed in the former (Fig. 6h). Likewise, a reduction in the number of KI67-positive cells was observed in the LHT7-treated group compared to the control group, demonstrating a decrease of 78.66% (Fig. 6i). Moreover, the condition of the mice improved with LHT7 treatment (Fig. 6j) and the survival rate increased compared to the control group (Fig. 6k). Taken together, these findings demonstrate that LHT7 effectively inhibits the growth of glioblastoma via dual mechanisms of anti-angiogenesis and anti-proliferation in an orthotopic mouse model.

## Discussion

Anti-angiogenic therapy has been a promising approach to the treatment of brain tumors. Anti-angiogenic agents target the blood vessels that support tumor growth, leading to a decreased blood supply and reduced tumor growth. Several clinical trials have been conducted to evaluate the efficacy and safety of anti-angiogenic drugs for the treatment of brain tumors, including glioblastoma, the most common and aggressive primary brain tumor [33]. Some of the most promising results have been seen with bevacizumab, an antibody that blocks the activity of VEGF, a protein involved in the development of blood vessels. However, despite promising results, anti-angiogenic therapy faces several challenges and limitations. One of the main challenges is the development of resistance to anti-angiogenic drugs, which can lead to tumor progression despite treatment [34]. Additionally, the effects of anti-angiogenic therapy can be limited by the difficulty in delivering drugs to the tumor site through the BBB [35, 36]. Another limitation is that anti-angiogenic therapy may not be effective in all patients, and some patients may experience adverse side effects. In addition, the use of anti-angiogenic therapy in combination with other treatments, such as chemotherapy and radiation therapy, can lead to increased toxicity and decreased efficacy. Overall, anti-angiogenic therapy remains a promising approach for the treatment of brain tumors, but further research is needed to optimize treatment strategies and improve outcomes for patients.

**Scheme 1:**
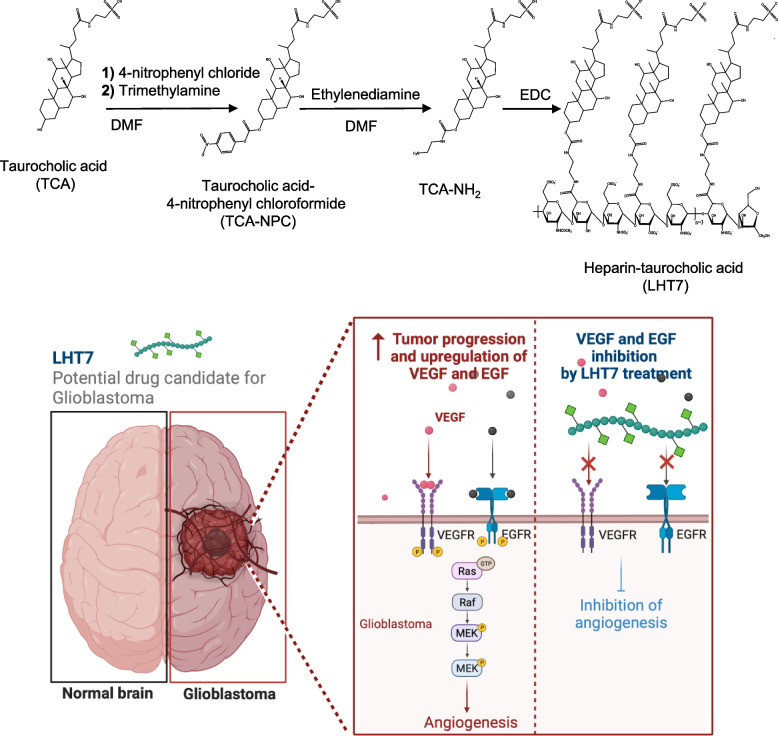
A comprehensive illustration of LHT7 synthesis and its mechanism of action in anti-angiogenic glioblastoma therapy

In this study, we aimed to address these challenges by evaluating the efficacy of LHT7, an anti-angiogenic drug, as a potential substitute for Avastin and as a novel strategy for glioblastoma treatment. Our investigation confirmed that LHT7 has anti-angiogenic and anti-proliferation effects due mainly to dephosphorylation of the ERK protein and reduced VEGF expression in glioblastoma cells and human umbilical vein endothelial cells (Scheme 1). The study also showed that LHT7 treatment decreased the proliferation of U87MG cells, which did not respond to over-treatment with growth factors. The study is the first to evaluate the anti-angiogenic and anti-proliferation effects of LHT7 in an orthotopic glioblastoma mouse model. The study confirmed that LHT7 inhibits angiogenesis ex vivo by decreasing VEGF expression in U87MG cells and affecting angiogenesis carried out by a rat aortic ring assay. The study also showed that the BBB is degraded in tumors of a certain size, and that LHT7 treatment (5 mg/kg) started 4 weeks after U87MG cell injection produced the most significant anti-angiogenic and anti-proliferation effects.

In conclusion, our study presents LHT7 as a highly promising and advanced alternative to existing anti-angiogenic therapies, such as Avastin, in the treatment of glioblastoma. Through rigorous quantitative comparisons with past methods reported in the literature, we have clearly demonstrated the superiority and advanced features of LHT7. Unlike previous approaches that struggled to effectively control tumor growth [37], LHT7 exhibited remarkable anti-angiogenic and anti-proliferative effects, even in U87MG cells that were unresponsive to overstimulation with growth factors. This ability to overcome treatment resistance sets LHT7 apart as a game-changing solution for patients who may have experienced limited benefits from other therapies. Moreover, our comprehensive evaluation using an orthotopic glioblastoma mouse model and ex vivo experiments, including the rat aortic ring assay, provided robust evidence of LHT7's exceptional anti-angiogenic efficacy. It outperformed other reported methods, clearly establishing LHT7 as a front-runner in inhibiting angiogenesis and thus depriving tumors of the blood supply necessary for their growth and survival. Notably, the optimal timing of LHT7 treatment, initiated four weeks after U87MG cell injection, showcased its ability to deliver maximum anti-angiogenic and anti-proliferative impact. This strategic approach further solidifies LHT7's position as an advanced and highly effective therapeutic agent, presenting a potential breakthrough in glioblastoma treatment.

In light of the promising anti-angiogenic and anti-proliferative effects observed with LHT7 treatment in our study, it is essential to acknowledge potential challenges associated with its repeated administration. Concerns include the development of drug resistance over time, cumulative toxicities, and possible interactions with standard treatments like chemotherapy and radiation therapy. Moreover, continuous evaluation of blood–brain barrier (BBB) permeability is crucial. To address these issues, future investigations should focus on understanding LHT7's long-term effects, exploring combination therapies, and optimizing dosing schedules. Preclinical studies and clinical trials will be vital in ensuring the safe and effective use of LHT7 as an anti-angiogenic therapy for glioblastoma.

## Conclusion

In conclusion, this study provides evidence of the potential efficacy of LHT7 as a promising anti-angiogenic drug for treatment of glioblastoma. The findings suggest that LHT7 inhibits angiogenesis and proliferation of glioblastoma cells and human umbilical vein endothelial cells by reducing VEGF expression and dephosphorylating the ERK protein. The study also demonstrated that LHT7 treatment inhibits angiogenesis in an orthotopic glioblastoma mouse model and reduces tumor growth, with the greatest effects observed when treatment was initiated 4 weeks after U87MG cell injection. Moreover, the study revealed that the BBB is disrupted in tumors of a certain size, which could facilitate the delivery of LHT7 to the tumor site. Although further studies are needed to optimize the treatment strategy and improve outcomes, the results of this study suggest that LHT7 has potential as an anti-angiogenic and anti-proliferative drug for the treatment of glioblastoma**.**

## Data Availability

The datasets used and/or analyzed during the current study are available from the corresponding author on reasonable request.

## Abbreviations

LHT7: Heparin-taurocholate conjugate
BCNU: 1,3-Bis(2-chloroethyl)-1-nitrosourea
MMR: Mismatch repair
DMF: N,N-dimethylformamide
4-NPC: 4-Nitrophenylchloroformate
BBB: Blood brain barrier
HUVEC: Human umbilical vein endothelial cells
MGMT: O^6^-methylguanine-DNA methyltransferase
VEGF: Vascular endothelial growth factor
VEGFR2: Vascular endothelial growth factor receptor type 2
PDGF: Platelet-derived growth factor
b-FGF: Fibroblast growth factor-basic
UFH: Unfractionated heparin
HSGAG: Highly sulfated glycosaminoglycan
LMWH: Low-molecular-weight heparin
GBM: Glioblastoma multiforme
NRP1: Neutrophilin-1
EPR: Enhanced permeability and retention
MFI: Mean fluorescence intensity
